# Charity coverage and decreased COVID-19 mortality among uninsured patients in Texas: A retrospective cohort study

**DOI:** 10.1371/journal.pone.0330533

**Published:** 2025-09-10

**Authors:** Anisha P. Ganguly, John T. Battaile, Michael Harms, Shannon Murray, Tami Gurley, Robert W. Haley, Mamta K. Jain, Kavita P. Bhavan

**Affiliations:** 1 Department of Medicine, University of North Carolina at Chapel Hill, Chapel Hill, North Carolina, United States of America; 2 Center of Innovation and Value, Parkland Health, Dallas, Texas, United States of America; 3 Department of Internal Medicine, University of Texas Southwestern Medical Center, Dallas, Texas, United States of America; 4 O’Donnell School of Public Health, University of Texas Southwestern Medical Center, Dallas, Texas, United States of America; University of Missouri School of Medicine, UNITED STATES OF AMERICA

## Abstract

**Purpose:**

Decreased access to care and social drivers of health have been implicated in COVID-19 disparities. The objective of this study was to test the association between county-funded charity coverage (CFCC) and mortality among uninsured patients hospitalized with COVID-19 in a highly uninsured county.

**Methods:**

This retrospective cohort study compared electronic health record (EHR) data among uninsured patients hospitalized with COVID-19 in a high-volume safety-net health system in Dallas County, Texas between June 2020 and December 2021. Uninsured patients included CFCC recipients and self-pay patients. We compared mortality over 180 days of follow-up using Cox proportional hazards models, adjusting for gender, age, race/ethnicity, and co-morbidities. Additional outcomes included 90-day mortality, need for mechanical ventilation, and intubation within 24 hours of presentation.

**Results:**

Among 2,047 patients, 47.0% received CFCC and 53.0% were self-pay. Overall, CFCC patients were older, more likely Hispanic, and had more diagnosed co-morbidities. CFCC patients had decreased adjusted mortality compared to self-pay (aHR 0.61, 95% CI [0.45 to 0.82], p < 0.01), with an absolute risk reduction of 3.3% and a number needed to treat (NNT) with CFCC of 30.4 (95% CI 21.4–66.6). CFCC was associated with lower 90-day mortality compared to self-pay (OR = 0.64, 95% CI [0.45–0.92], p = 0.01), despite similar need for ventilation. Intubation within 24 hours of presentation was lower for CFCC compared to self-pay (OR = 0.46, 95% CI [0.22–0.93], p = 0.03).

**Conclusions:**

CFCC was associated with decreased mortality among the uninsured hospitalized with COVID-19. The NNT for CFCC to prevent 1 death among uninsured patients was similar to that for standard medications to treat COVID-19. These findings support expanding coverage to improve COVID-19 outcomes.

## Introduction

The COVID-19 pandemic has exposed marked inequities in health outcomes and access to healthcare in the United States (US). With more than 100 million cases diagnosed and 1.1 million deaths [1], the COVID-19 pandemic has revealed significant racial and socioeconomic disparities [2–5]. Disparities have been demonstrated in exposure risk related to essential worker status, utilization of public infrastructure, and population density [6–8], baseline medical vulnerability, and increased risk for severe COVID-19, including mortality [9–11].

Texas is the largest Medicaid non-expansion state in the US with a 19.9% uninsured rate, nearly 2.5 times the national average [12,13]. Located in Dallas County, Parkland Health (“Parkland”) serves as the safety-net for more than 2.6 million people, including half a million uninsured [14]. Parkland provides county-funded charity coverage (CFCC) for uninsured Dallas County residents with less restrictive income eligibility than requirements for Medicaid in expansion states [12,15]. Previous research has indicated that charity coverage improves health outcomes among the uninsured. For example, a recent study in the Kaiser Permanente health system showed that charitable financial assistance increased ambulatory care utilization and screening and management of diabetes [16]; similarly, an analysis in Washington and Florida suggested that increased health department financial assistance improved birth outcomes in low-income counties [17]. Studies comparing health outcomes among recipients of charity coverage like CFCC compared to self-pay patients, particularly within highly uninsured populations, remain limited.

Like other safety-net hospitals [18], Parkland experienced historically high inpatient and critical care volumes during the height of the pandemic. Uninsured patients without prior contact with the healthcare system along with patients receiving CFCC were hospitalized with COVID-19. Prior research has characterized racial and socioeconomic disparities in COVID-19 [3,19,20]. Lower rates of insurance coverage are associated with increased COVID-19 incidence, hospitalizations, intensive care unit (ICU) admissions, and mortality [21]. The role of CFCC among uninsured populations hospitalized with COVID-19 remains unexplored. The purpose of this study was to evaluate the differences in COVID-19 mortality between CFCC and self-pay patients.

## Materials and methods

### I. Setting

Parkland is a safety-net health system comprised of an 882-bed hospital, 16 primary care clinics, 11 women’s health centers, 4 medical mobile vans, and numerous specialty clinics amounting to more than 1 million outpatient visits annually. More than 50% of the Parkland patient population identifies as Hispanic/Latinx and approximately 30% identify as Black [22]. Parkland serves a majority uninsured population (approximately 50%), with 35% of patients receiving Medicaid [22]. CFCC is available to all Dallas County residents at or below 250% of the federal poverty level (FPL), regardless of citizenship status [15]. CFCC is not insurance; it provides access to primary care, specialty care, comprehensive pharmacy coverage, and coverage for hospitalization within the health system. Patients ineligible for CFCC due to income higher than 250% FPL or residence out of Dallas County are categorized as self-pay and may receive care from the health system on a discount plan tiered based on patient income. As the only academic safety-net health system in Dallas, self-pay patients continue to seek care at Parkland over other hospitals even without receipt of CFCC [23]; in FY2023, 8.9% of Parkland patients were self-pay [24].

### II. Study population and time period

The study population included all patients aged 18 and older who were hospitalized with COVID-19 from June 2020 to December 2021. The initial three months of the pandemic were excluded due to practice of early intubation for COVID-19 infection, a critical care practice in which patients were intubated prior to standard clinical presentation of respiratory compromise, often within 24 hours of transfer to intensive care [25,26]. As the practice of early intubation may bias representation of severity of COVID-19 infection, hospitalizations during this time period of clinical practice were excluded. COVID-19 hospitalization was defined by diagnosis codes. Pregnant women were excluded from the dataset, as pregnancy is a Medicaid-qualifying condition in Texas [27], and criteria for COVID-19 hospitalization in pregnancy differed from the non-pregnant population [28]. Study time period was segmented into the following waves of the pandemic based on national case rates and other research exploring temporal waves in COVID-19 variants: pre-vaccine (June 1, 2020 to May 31, 2021, end of peak vaccination), Delta variant wave (June 1, 2021-October 31, 2021), and Omicron variant wave (November 1, 2021 to December 31, 2021, end of study period) [1,29,30].

### III. Data source and outcomes

Clinical and healthcare utilization data associated with COVID-19 hospitalization encounters and encounters for the year prior to and after COVID-19 hospitalization were extracted from the electronic health record (EHR). EHR data were limited to encounters within the health system; encounters from neighboring hospitals and clinics were not accessible. Data sources from the EHR included demographic data, diagnosis codes, clinical flowsheets, laboratory data, medications, and billing data associated with each COVID-19 hospital encounters. Data extraction occurred from August to September 2022.

The primary outcome of interest was mortality rate after COVID-19 hospitalization over 180 days of follow-up. Mortality was determined by death recorded in the EHR or completed clinical encounters after 180 days of follow-up indicating a patient’s survival. To mitigate potential bias introduced by lack of follow-up after discharge, differentially more common among self-pay patients, patients were censored based on the last known encounter date before the follow-up time period.

Secondary outcomes included 90-day all-cause mortality, need for mechanical ventilation at any point during hospitalization, and need for intubation within 24 hours of initial presentation. Need for intubation within 24 hours was examined as a secondary outcome to reflect severity of disease upon initial presentation warranting urgent need for mechanical ventilation.

### IV. Study design and analytic method

We conducted a retrospective cohort study of COVID-19 hospitalization encounters among CFCC and self-pay patients. Payer status was defined by billing data associated with the hospital encounter. Many patients initially categorized under Medicare were recategorized as uninsured if they were uninsured patients who were billed as Medicare under the Health Resources and Services Administration (HRSA) COVID-19 Uninsured Program [31].

Demographic and baseline characteristic data were compared among payer cohorts using the χ2 test for categorial data. For continuous variables, the Kruskal-Wallis test was used for non-parametric data and two-sample t-test for parametric data.

Cox proportional hazards survival analysis compared mortality after COVID-19 hospitalization, controlling for clinically significant covariates in COVID-19 infection, including gender, age, race/ethnicity, and comorbidities. Race/ethnicity was categorized in five groups, Hispanic, non-Hispanic Black, non-Hispanic Asian, non-Hispanic white, and other. EHR race/ethnicity information is self-reported by patients, either electronically in the patient portal or verbally to administrative staff during registration. Comorbidities included in original data extraction encompassed the Centers for Disease Control and Prevention (CDC) list of medical conditions predisposing to COVID-19 [32]. Chronic lung disease, diabetes, end-stage kidney disease, heart conditions, and liver disease were discretely examined as individual predisposing comorbidities. Remaining comorbidities were included in the model as a continuous variable. Survival analysis was extended to 180 days of follow-up. Number needed to treat with CFCC was calculated from the adjusted hazard ratio (aHR) at a given time using the following formula,


$$\frac{1}{{\left[S_{c}(t)\right]}^{h}-S_{c}(t)}$$

where S_c_(t)=survival probability of control and h=aHR [33].

Multivariable logistic regression was used to assess the association of receiving CFCC and secondary outcomes while adjusting for the same covariates used in survival analysis. Statistical significance was based on a two-sided alpha level of 0.05. All statistical analysis was performed with Python 3.7 (Python Software Foundation).

### V. Ethics statement

This study was approved by the University of Texas Southwestern Institutional Review Board and followed the Strengthening the Reporting of Observational Studies in Epidemiology (STROBE) reporting guidelines. For this retrospective study, patient consent was not obtained, which was approved by the above Institutional Review Board. The authors had access to identifiable patient information during this study. Data was accessed between March 9, 2022 through July 17, 2023.

## Results

The study population was comprised of 3,650 patients hospitalized with COVID-19, of whom 340 (9.3%) were commercially insured, 553 (15.2%) received Medicaid, 710 (19.4%) received Medicare, and 2,047 (56.1%) were uninsured (S1 Table). Among 2,047 uninsured patients, 962 (47.0%) received CFCC, and 1,085 (53.0%) were self-pay. Baseline characteristics of the CFCC and self-pay patients are provided in Table 1. There were notable differences in most baseline parameters: self-pay patients were more likely to be male and younger, while CFCC patients were more likely to be older, identify as Hispanic, and speak Spanish as their preferred language.

**Table 1:** Baseline characteristics of CFCC and self-pay patients hospitalized with COVID-19.

		Total Uninsured N = 2,047	CFCC N = 962	Self-Pay N = 1,085	p-value
Gender	Female	823 (40.2)	446 (46.4)	377 (34.7)	<0.001
	Male	1224 (59.8)	516 (53.6)	708 (65.3)	
Age	18-34	294 (14.4)	85 (8.8)	209 (19.3)	<0.001
	35-49	754 (36.8)	346 (36.0)	408 (37.6)	
	50-64	812 (39.7)	432 (44.9)	380 (35.0)	
	65-79	134 (6.5)	67 (7.0)	67 (6.2)	
	80+	53 (2.6)	32 (3.3)	21 (1.9)	
Race/Ethnicity	Hispanic	1530 (74.7)	757 (78.7)	773 (71.2)	<0.001
	Non-Hispanic Black	311 (15.2)	138 (14.3)	173 (15.9)	
	Non-Hispanic White	148 (7.2)	51 (5.3)	97 (8.9)	
	Asian	22 (1.1)	11 (1.1)	11 (1.0)	
	Other	36 (1.8)	5 (0.5)	31 (2.9)	
Language	English	796 (38.9)	312 (32.4)	484 (44.6)	<0.001
	Spanish	1220 (59.6)	633 (65.8)	587 (54.1)	
	Other	31 (1.5)	17 (1.8)	14 (1.3)	
Smoking	Never smoker	1461 (71.4)	711 (73.9)	750 (69.1)	<0.001
	Former smoker	326 (15.9)	175 (18.2)	151 (13.9)	
	Current smoker	217 (10.6)	69 (7.2)	148 (13.6)	
	Unknown	43 (2.1)	7 (0.7)	36 (3.3)	
County of residence	Dallas	1900 (92.8)	946 (98.3)	954 (87.9)	<0.001
	Other	147 (7.2)	16 (1.7)	131 (12.1)	
COVID wave	Pre-vaccine	1368 (66.8)	695 (72.2)	673 (62.0)	<0.001
	Delta	585 (28.6)	230 (23.9)	355 (32.7)	
	Omicron	94 (4.6)	37 (3.8)	57 (5.3)	
Predisposing comorbidities	Diabetes	890 (43.5)	490 (50.9)	400 (36.9)	<0.001
	Heart disease	1209 (59.1)	663 (68.9)	546 (50.3)	0.004
	End-stage kidney disease	49 (2.4)	33 (3.4)	16 (1.5)	0.003
	Chronic lung disease	221 (10.8)	118 (12.3)	103 (9.5)	0.04
	Chronic liver disease	267 (13.0)	131 (13.6)	136 (12.5)	0.468
Number of comorbidities**	0-1	654 (31.9)	227 (23.6)	427 (39.4)	<0.001
	2-3	672 (32.8)	309 (32.1)	363 (33.5)	
	4-5	587 (28.7)	339 (35.2)	248 (22.9)	
	6-7	125 (6.1)	81 (8.4)	44 (4.1)	
	8+	9 (0.4)	6 (0.6)	3 (0.3)	
Known PCP	Yes	758 (37.0)	548 (57.0)	210 (19.4)	<0.001
	No	1289 (63.0)	414 (43.0)	875 (80.6)	
Number of outpatient visits in the year prior to index admission	0	1153 (56.3)	304 (31.6)	849 (78.2)	<0.001
	1	103 (5.0)	57 (5.9)	46 (4.2)	
	2-4	193 (9.4)	115 (12.0)	78 (7.2)	
	5+	598 (29.2)	486 (50.5)	112 (10.3)	

*Values in parenthesis reflect percentage of patients.

**Comorbidities included: cancer, chronic kidney disease and end-stage kidney disease, chronic lung disease, cystic fibrosis, neurologic disorders, diabetes, heart conditions, HIV, hypertension, immunodeficiencies, liver disease, obesity, sickle cell disease or thalassemia, smoking, transplant, stroke, substance use, and tuberculosis.

CFCC hospitalizations were more likely to occur in the pre-vaccine era; self-pay patients had a higher percentage of hospitalizations during the Delta and Omicron waves of the pandemic than those with CFCC. CFCC patients had more diagnosed comorbidities and were more likely to have a known primary care provider (PCP), 56.8%, compared to 19.4% (p < 0.001) among self-pay patients. CFCC had a higher rate of outpatient visits in the health system in the year prior to the index admission; 50.5% of CFCC patients had ≥ 5 outpatient visits, while 78.2% of self-pay patients had none.

CFCC and self-pay patients had similar receipt of COVID-directed therapies including dexamethasone, remdesivir, and monoclonal antibody treatment (p = 0.67). Discharge disposition greatly differed across the two groups (p < 0.001). CFCC patients were more likely to discharge to home/self-care (87.5% compared to 81.5% among the self-pay patients) or an inpatient rehabilitation facility/hospital transfer (2.9% compared to 1.6% among the self-pay patients); self-pay patients were more likely to leave against medical advice (2.4% vs 0.6%).

**Fig 1:**
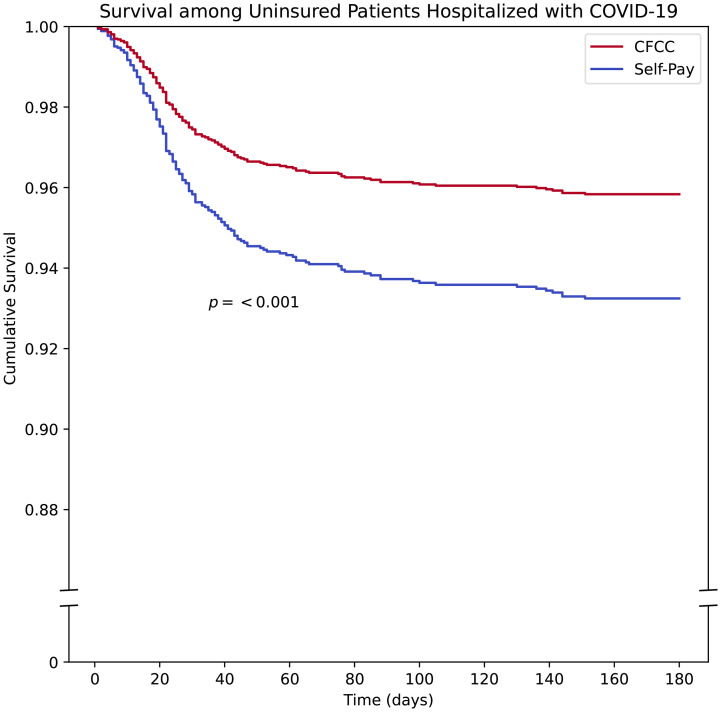
Cox proportional hazards model of mortality among patients hospitalized with COVID-19 over 180 days of follow-up.

We explored the relationship of CFCC and all-cause mortality COVID using Cox proportional hazards regression analysis, adjusting for gender, age, race/ethnicity, and co-morbidities. Unadjusted, the 180-day mortality rate among patients receiving CFCC was 9.0%, compared to 8.7% among self-pay patients (p = 0.76). Results of survival analysis over 180 days of follow-up are shown in Table 2 and Fig 1. The adjusted mortality rate was lower for CFCC patients compared to the self-pay patients (aHR 0.61, 95% CI [0.45 to 0.82], p < 0.01), with an absolute risk reduction of 3.3% and a number needed to treat (NNT) with CFCC to prevent 1 death of 30.4 (95% CI 21.4–66.6). Significant covariates included female gender, older age, and number of comorbidities. Hispanic ethnicity was independently associated with decreased survival. In a sensitivity analysis stratifying patients by age, mortality rate was consistently lower for CFCC patients compared to self-pay patients among both patients aged 18−49 (aHR 0.49, 95% CI [0.27–0.9], p = 0.02) and patients 50 and older (aHR 0.65, 95% CI [0.46–0.92], p = 0.02).

**Table 2:** Multivariable Cox proportional hazards regression analysis for 180-day mortality among uninsured patients hospitalized with COVID-19.

		Adjusted Hazard Ratio (OR 95% CI)	p-value
Payer Status	CFCC	0.61 (0.45, 0.82)	<0.01
	Self-pay	ref	
Gender	Female	0.57 (0.42, 0.78)	<0.01
	Male	ref	
Age	18-34	ref	0.08 0.01 <0.01 <0.01
	35-49	2.32 (0.91, 5.92)	
	50-64	3.50 (1.41, 8.72)	
	65-79	9.51 (3.69, 24.5)	
	80+	10.51 (3.62, 30.5)	
Race/Ethnicity	Hispanic	2.24 (1.04, 4.85)	0.04 0.23 0.60 0.74
	Non-Hispanic Black	1.66 (0.72, 3.82)	
	Non-Hispanic White	ref	
	Asian	1.53 (0.31, 7.49)	
	Other	0.7 (0.08, 5.75)	
Number of remaining comorbidities	---	1.23 (1.05, 1.43)	0.01
Predisposing comorbidities	Diabetes	1.18 (0.87, 1.60)	0.29
	Heart disease	4.19 (2.37, 7.41)	<0.01
	End-stage kidney disease	1.12 (0.54, 2.30)	0.76
	Chronic lung disease	1.73 (1.17, 2.54)	0.01
	Chronic liver disease	3.28 (2.39, 4.5)	<0.01

Secondary outcomes were further explored in a multivariable model (Table 3). After adjusting for gender, age, race/ethnicity, and comorbidities, CFCC recipients were found to have lower 90-day mortality than the self-pay patients (aOR 0.64, 95% CI [0.45–0.92], p = 0.014), consistent with the differences noted in survival analysis. As in the Cox regression model, female gender, older age and Hispanic ethnicity were associated with higher 90-day mortality, as were the number of comorbidities and specifically co-morbid heart disease, chronic lung disease, and chronic liver disease. The relationship between Hispanic ethnicity and CFCC was explored in a separate model including an interaction term, which was not found to be significantly associated with 90-day mortality (p = 0.75). There were no differences in need for mechanical ventilation between CFCC and self-pay patients (aOR 0.98, 95% CI [0.73–1.32], p = 0.91). CFCC patients had a lower rate of intubation within 24 hours of presentation (aOR 0.46, 95% CI [0.22–0.93], p = 0.003). Number of comorbidities was similarly associated with increased need for intubation within 24 hours (p < 0.001).

**Table 3:** Multivariable logistic regression for critical care outcomes among uninsured patients hospitalized with COVID-19.

		90-day mortality (aOR 95% CI)	p-value	Need for mechanical ventilation (aOR 95% CI)	p-value	Intubation within 24 hours (aOR 95% CI)	p-value
Payer Status	CFCC	0.64 (0.45-0.92)	0.01	0.98 (0.73-1.32)	0.91	0.46 (0.22-0.93)	0.03
	Self-pay	ref		ref		ref	
Gender	Female	0.51 (0.35-0.74)	<0.001	0.53 (0.39-0.72)	<0.001	0.82 (0.41-1.65)	0.58
	Male	ref		ref		ref	
Age	18-34	ref		ref		ref	
	35-49	2.50 (0.94-6.61)	0.07	1.44 (0.80-2.61)	0.23	1.02 (0.35-3.00)	0.97
	50-64	4.11 (1.58-10.65)	0.004	1.96 (1.09-3.51)	0.02	0.85 (0.29-2.54)	0.78
	65-79	11.98 (4.33-33.14)	<0.001	3.89 (1.96-7.74)	<0.001	0.41 (0.07-2.50)	0.34
	80+	11.72 (3.63-37.86)	<0.001	1.69 (0.62-4.64)	0.31	1.58 (0.27-9.35)	0.62
Race/ Ethnicity	Hispanic	3.30 (1.36-8.03)	0.009	1.60 (0.89-2.86)	0.12	0.68 (0.27-1.67)	0.40
	Non-Hispanic Black	2.38 (0.91-6.23)	0.08	0.83 (0.42-1.62)	0.58	0.18 (0.04-0.74)	0.02
	Non-Hispanic White	ref		ref		ref	
	Asian	1.66 (0.27-10.35)	0.59	1.4 (0.34-5.85)	0.64	ref*	
	Other	0.68 (0.06-7.15)	0.75	0.78 (0.13-4.60)	0.78	2.34 (0.22-25.0)	0.48
Number of comorbidities	---	1.25 (1.04-1.50)	0.02	1.6 (1.37-1.87)	<0.001	2.23 (1.61-3.1)	<0.001
Predisposing comorbidities	Diabetes	1.34 (0.94-1.91)	0.11	1.30 (0.96-1.75)	0.08	1.32 (0.65-2.65)	0.44
	Heart disease	5.45 (2.96-10.05)	<0.001	2.89 (1.93-4.34)	<0.001	2.13 (0.88-5.15)	0.10
	End-stage kidney disease	0.73 (0.27-1.98)	0.54	1.84 (0.92-3.71)	0.09	1.84 (0.39-8.57)	0.44
	Chronic lung disease	2.00 (1.25-3.18)	0.004	1.33 (0.87-2.03)	0.19	1.45 (0.60-3.53)	0.41
	Chronic liver disease	4.25 (2.90-6.22)	<0.001	3.32 (2.39-4.61)	<0.001	1.67 (0.77-3.59)	0.19

*Due to lower unadjusted rates of intubation across groups, Asian patients were consolidated with Non-Hispanic White patients in the reference group for the outcome of intubation within 24 hours.

## Discussion

In our safety-net health system, uninsured patients receiving CFCC hospitalized for COVID-19 had a nearly 40% lower mortality rate compared to self-pay patients. The NNT for CFCC to prevent a death among uninsured patients hospitalized with COVID-19 was 30. Our study provides supportive evidence for expanded coverage as a life-saving benefit. Few factors have been associated with a positive effect on COVID-19 mortality. To contextualize the mortality difference demonstrated in this analysis, compare the NNT of 30.4 for CFCC to that of dexamethasone, one of the mainstays of COVID-19 management: 28.0–37.4 [34,35].

Notably, the coverage disparity demonstrated in mortality was not attributable to severity of COVID-19 infection, as illustrated by similar need for mechanical ventilation [36]. In adjusted analyses, CFCC and self-pay patients had similar rates of mechanical ventilation, suggesting similar threshold of severity of infection progressing to intubation. Differences in mortality may be attributed to differences preceding hospitalization. Despite similar need for ventilation, differences such as baseline cardiopulmonary risk, which has been associated with extubation failure and ICU mortality [37,38], may have led to differences in mortality downstream of ICU course.

This hypothesis is supported by our findings in differences of diagnosed comorbidities and known PCP. Although CFCC patients had a higher rate of known diagnosed comorbidities, self-pay patients may have had a higher rate of undiagnosed, and therefore uncontrolled, comorbidities. CFCC patients were 3 times more likely to have a known PCP and had a higher rate of ambulatory visits in the year prior to hospitalization, demonstrating engagement in streamlined outpatient care within the same system.

An additional factor that could have contributed to higher mortality among the COVID-19 self-pay patients is the role of vaccination. Self-pay patients comprised a greater distribution of hospitalizations in the post-vaccine era, which could indicate lower vaccination rates in this population. Lower vaccination rates similarly demonstrate inequities in access to preventive care. Uninsurance has been associated with COVID vaccine hesitancy [39], and lack of a PCP, noted in the self-pay population, has been associated with decreased influenza vaccination [40].

Lastly, self-pay patients were more than twice as likely to undergo intubation within 24 hours of presentation. Prior research has demonstrated deferral of care among the uninsured due to risk for financial harm and burden of medical debt [41,42], even in the context of catastrophic health events in emergent settings [43]. In our study, adjusted need for mechanical ventilation was similar across coverage cohorts, however need for precipitous intubation was higher among the self-pay patients. This suggests similar *threshold of severity* across the two coverage cohorts, but a difference in *timepoint* when patients sought medical care, which may have contributed to increased mortality among the self-pay patients. Deferral of care among self-pay patients is further supported by lower 30-day readmission, which may suggest increased risk of mortality at home after discharge.

Our single-center study expands upon prior research on COVID-19 disparities. The literature has demonstrated racial/ethnic and socioeconomic disparities in every aspect of COVID-19 infection: exposure, hospitalization, and mortality [2,3,19]. Characterization of racial/ethnic disparities requires a critical, nuanced assessment of structural factors that drive inequitable COVID-19 outcomes, including access to care and coverage. As discussed by Chowkwanyun and Reed, simplistic associations of race/ethnicity and COVID-19 outcomes can lend credence to biological explanations in racial health disparities, stigmatization of minoritized populations, and lack of locoregional nuance in distribution of racial/ethnic disparities [44]. Our study population is comprised of largely Hispanic and Black patients served within a single safety-net health system in a highly uninsured county [14,45]. Contrary to other findings [46], Black race was not associated with worse COVID-19 outcomes in our system. Conversely, Hispanic ethnicity was independently associated with COVID-19 mortality. These findings underscore the imperative for nuanced local analyses of racial/ethnic disparities and structural drivers in COVID-19 disparities, including coverage.

Our study findings support recent data at the national level measuring the impact of fragmented healthcare coverage on pandemic outcomes. A 2022 observational study utilizing CDC surveillance data demonstrated an association between lower health insurance rates and COVID-19 cases, hospitalizations, and deaths, persistent after stratification across age, gender, race, and state [21]. The study further estimated 26% avoidable COVID-19 mortality due to lack of insurance, a figure similar to the findings reported in our study. Similar analyses estimate that in the year 2020 alone, more than 200,000 deaths could have been avoided under a single-payer universal healthcare system [47,48].

Our study characterizes disparities at a local level within the fragmented national healthcare system, in a highly uninsured urban county. In our environment, CFCC is the closest approximation to expanded coverage. Current Medicaid eligibility in Texas is limited to US citizens who are disabled, pregnant or up to 12 months postpartum, children, or above the age of 65, and with income requirements more restrictive than that under Medicaid expansion [49]. In contrast, CFCC is extended to all Dallas County residents, regardless of age, qualifying diagnosis, or citizenship, with less restrictive income requirements. CFCC extends beyond uncompensated care in the emergency department (ED) or inpatient settings covered under the Emergency Medical Treatment and Active Labor Act (EMTALA) to include comprehensive primary and specialty care [15]. As demonstrated in national studies on county spending on social services and health outcomes [50,51], increased county investment was associated with improved health outcomes during the COVID-19 pandemic.

This study contains several limitations. EHR data was limited to encounters within our safety-net health system. Neighboring hospitals may be more utilized by the self-pay patients than those with CFCC, as CFCC is solely administered by our health system, whereas the self-pay patients may seek uncompensated care anywhere. Conversely, the single-center nature of this study is also a strength, in that we were able to present more granular clinical data based on multiple data sources integrated within the system EHR. Use of institutional data was additionally necessary to present nuanced demographic and clinical data among self-pay patients, a population under-characterized in claims data analyses. We were unable to measure out-of-hospital mortality using our EHR data; given the disproportionate lack of follow-up data in the self-pay population, we utilized a censoring approach based on last encounter date in survival analysis. Nonetheless, increased censoring among self-pay patients served to bias towards the null, and we hypothesize that out-of-hospital mortality would further widen the observed disparity in survival. Lastly, this analysis is limited to a single safety-net health system in a geographic area with unique demographic composition and structural determinants that may limit the generalizability of our findings.

## Conclusion

In this observational study, CFCC was associated with a significant reduction in mortality among uninsured patients hospitalized with COVID-19. These findings reveal a sizable opportunity to improve clinical outcomes with coverage expansion, as simulated by CFCC in a highly uninsured environment. These disparities underscore the opportunity for improved public health with expanded coverage.

## Supporting information

S1 Table Baseline characteristics of patients hospitalized with COVID-19 by Payer status, n = 3,650. (DOCX)
